# Efeitos da Suplementação de Vitamina D sobre Parâmetros Hemodinâmicos Centrais e Sistema Nervoso Autônomo em Indivíduos com Obesidade ou Sobrepeso

**DOI:** 10.36660/abc.20230678

**Published:** 2024-04-26

**Authors:** Adriana C. C. Faria, Caroline Lyra Moreira, Michelle Rabello da Cunha, Samanta Mattos, Wille Oigman, Mario Fritsch Neves

**Affiliations:** 1 Departamento de Clínica Médica Universidade do Estado do Rio de Janeiro Rio de Janeiro RJ Brasil Departamento de Clínica Médica, Universidade do Estado do Rio de Janeiro, Rio de Janeiro, RJ – Brasil; 2 Departamento de Nutrição Aplicada Instituto de Nutrição Universidade do Estado do Rio de Janeiro Rio de Janeiro RJ Brasil Departamento de Nutrição Aplicada, Instituto de Nutrição, Universidade do Estado do Rio de Janeiro, Rio de Janeiro, RJ – Brasil

**Keywords:** Vitamina D, Sistema Nervoso Autônomo, Hemodinâmica

## Abstract

**Fundamento:**

Estudos prévios têm sido inconsistentes em demonstrar efeitos cardiovasculares benéficos da suplementação de vitamina D.

**Objetivo:**

Avaliar efeitos da suplementação de vitamina D3 sobre parâmetros hemodinâmicos centrais e atividade autonômica em indivíduos obesos/sobrepeso e baixos níveis de vitamina D (<30ng/dl).

**Métodos:**

Ensaio clínico prospectivo, randomizado, duplo-cego (NCT 05689632), adultos 40-65 anos com índice de massa corporal ≥25<40 kg/m^2^. Hemodinâmica central avaliada por método oscilométrico (Mobil-O-Graph®), variabilidade da frequência cardíaca utilizando frequencímetro Polar (software Kubios^®^). Os pacientes (n=53) receberam placebo no grupo controle (CO, n=25) ou vitamina D3 (VD, n=28) 7000 UI/dia, avaliados antes (S0) e após 8 semanas (S8) com nível de significância de 0,05.

**Resultados:**

Os grupos foram homogêneos na idade (51±6 vs. 52±6 anos, p=0,509) e níveis de vitamina D (22,8±4,9 vs. 21,7±4,5ng/ml, p=0,590). Na S8, o grupo VD apresentou níveis significativamente maiores de vitamina D (22,5 vs. 35,6ng/ml, p<0,001). Apenas o grupo VD mostrou redução significativa da pressão arterial sistólica (PAS; 123±15 vs. 119±14mmHg, p=0,019) e fosfatase alcalina (213±55 vs. 202±55mg/dl, p=0,012). O grupo CO mostrou elevação da pressão de aumento (AP: 9 vs. 12mmHg, p=0,028) e do índice de incremento (Aix: 26 vs. 35%, p=0,020), o que não foi observado no grupo VD (AP: 8 vs. 8mmHg, Aix: 26 vs. 25%, p>0,05). Grupo VD apresentou aumento no índice do sistema nervoso (iSN) parassimpático (-0,64±0,94 vs. -0,16±1,10, p=0,028) e no intervalo R-R (866±138 vs. 924±161ms, p=0,026).

**Conclusão:**

Nesta amostra, a suplementação diária de vitamina D durante oito semanas resultou em melhora dos níveis pressóricos, parâmetros hemodinâmicos centrais e do equilíbrio autonômico.

## Graphical abstract

**Figura Central:**
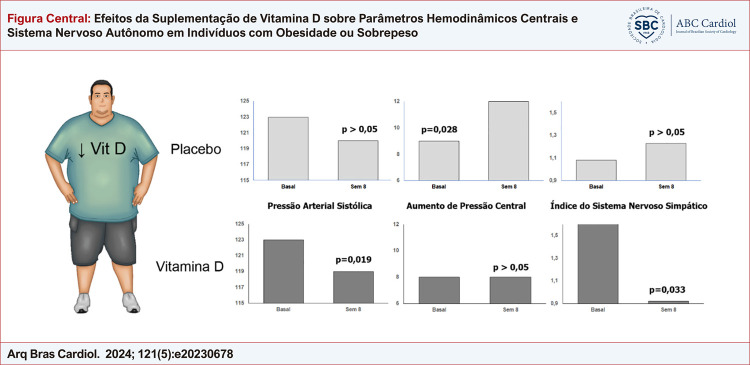
: Efeitos da Suplementação de Vitamina D sobre Parâmetros Hemodinâmicos Centrais e Sistema Nervoso Autônomo em Indivíduos com Obesidade ou Sobrepeso

## Introdução

A obesidade contribui diretamente para uma elevação do risco cardiovascular com aumento de mortalidade, independentemente de outros fatores de risco. Uma variedade de mecanismos pode modular esse efeito adverso, uma vez que existe considerável heterogeneidade metabólica e inflamatória entre indivíduos com obesidade e sobrepeso.^1-3^ A vitamina D é um hormônio esteroide essencial para regulação da saúde esquelética e concentrações de 25-hidroxivitamina D (25(OH)D) entre 20ng/ml e 50ng/ml (50–125 nmol/l) foram consideradas seguras para a manutenção da saúde esquelética na população geral.^4,5^ Nos últimos anos, evidências revelaram papéis funcionais adicionais da vitamina D (efeitos pleiotrópicos), ligando sua deficiência (25(OH)D < 20ng/dl) e estado subótimo (25(OH)D ≥ 20 e <30ng/dl) a várias doenças e fatores metabólicos desfavoráveis, como resistência à insulina, diabetes melito tipo 2 e doenças cardiovasculares (DCV).^6,7^ Estudos epidemiológicos demonstraram que a deficiência de vitamina D está associada a um risco aumentado de eventos cardiovasculares futuros e rigidez arterial.^8,9^

A dificuldade de manutenção de níveis adequados de vitamina D é altamente prevalente entre os indivíduos com excesso de peso, provavelmente por diluição volumétrica (lipossolubilidade) e por sequestro pelo tecido adiposo (reservatório) entre outras hipóteses.^10,11^ A resposta à suplementação de vitamina D é menor nestes indivíduos, sugerindo ajustes de dose ao tamanho corporal.^12^ A obesidade e a deficiência de vitamina D juntas promovem inflamação e induzem disfunção vascular através do aumento da secreção de fatores de vasoconstrição, importantes contribuintes para a inflamação vascular e ativação endotelial. Ambas se associam com pior saúde cardiovascular e são consideradas fatores de risco para alterações vasculares, metabólicas e pior desfecho clínico.^13,14^

Estudos observacionais sustentam a teoria fisiológica da suplementação de vitamina D visando melhorar parâmetros vasculares e eventos clínicos.^15^ Entretanto, os resultados dos ensaios clínicos randomizados (ECR) têm sido inconsistentes quanto ao efeito protetor. Alguns fatores, como populações heterogêneas e doses de suplementação insuficientes para alterar o estado prévio de vitamina D, podem ser relacionados com essas falhas.^14^ A inclusão de indivíduos com deficiência ou insuficiência de Vitamina D sérica, e a utilização de doses elevadas e seguras para suplementação de Vitamina D adotadas neste estudo seguem critérios recentes estipulados para realização de estudos com nutrientes.^16^

Até o presente momento não foram observados estudos visando melhora na variabilidade da frequência cardíaca (VFC) em pacientes com baixos níveis de vitamina D e excesso de peso. O objetivo deste estudo foi avaliar os efeitos da suplementação de vitamina D sobre o sistema cardiovascular através da avaliação da rigidez arterial e da atividade do sistema nervoso autônomo.

## Métodos

### População do estudo

Voluntários adultos, de ambos os gêneros, com idade entre 40 e 70 anos, índice de massa corporal (IMC) entre 25,0 e 39,9 Kg/m^2^ e níveis de 25(OH)D <30 ng/ml foram incluídos no estudo. Os critérios de exclusão foram diabetes melito, doença arterial periférica, coronária ou renal crônica, usuários de betabloqueadores ou suplementos contendo vitamina D, gestação ou câncer nos últimos 5 anos. O protocolo foi aprovado pelo Comitê de Ética em Pesquisa local (CAAE: 61044522.0.0000.5259) e todos os participantes leram e assinaram o Termo de Consentimento Livre e Esclarecido (TCLE). A pesquisa ocorreu de acordo coma a resolução nº 466/2012. O ensaio clínico foi registrado no Clinicaltrials.gov (NCT 05689632).

### Desenho do estudo e randomização

**Figura 1:**
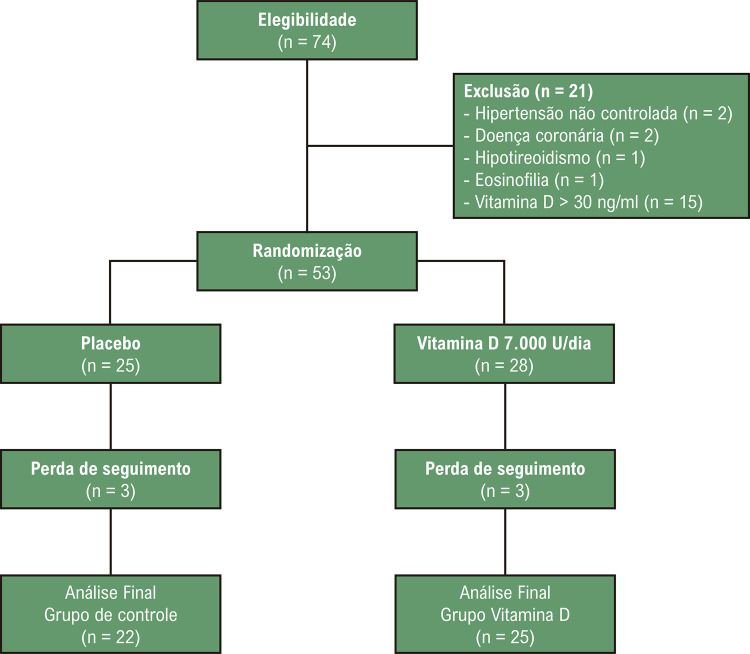
– Fluxograma do estudo.

Ensaio clínico prospectivo, randomizado, duplo-cego, controlado por placebo realizado na Clínica de Hipertensão Arterial e Doenças Metabólicas Associadas, no Hospital Universitário Pedro Ernesto, Universidade do Estado do Rio de Janeiro, Brasil. A Figura 1 apresenta o fluxograma do estudo com 53 participantes designados aleatoriamente, segundo plano de randomização (*wwww.randomization.com*), para receber placebo (triglicerídeos de cadeia média (TCM) 100mg) no grupo controle (CO, n=25) ou Vitamina D3 7.000UI e TCM 100mg no grupo intervenção (VD, n=28), diariamente após o almoço, durante 8 semanas. O ensaio foi duplo cego, para a equipe e os voluntários, que recebiam frascos sem rótulo, numerados, contendo o mesmo número de cápsulas idênticas em cor, formato e tamanho. Todas as cápsulas foram manufaturadas na mesma unidade (Centralfarma – Ipatinga, Minas Gerais, Brasil). A adesão foi avaliada pela porcentagem de cápsulas consumidas e uma taxa de adesão de 80% foi considerada satisfatória. Todos os exames foram realizados antes (S0) e após 8 semanas (S8) de intervenção.

### Avaliação pressórica, antropométrica e da composição corporal

As medidas da pressão arterial sistólica (PAS) e diastólica (PAD), cálculo da pressão arterial média (PAM), e frequência cardíaca (FC) foram obtidas por dispositivo digital calibrado (modelo HEM-705 CP, *OMRON Healthcare Inc*., Illinois), em posição sentada, no membro superior direito (MSD), após 5 minutos de repouso.

O peso foi aferido em balança digital com precisão de ± 0,1 kg (Filizola SA, São Paulo, SP, Brasil) e o IMC foi calculado pela fórmula: peso em quilogramas dividido pelo quadrado da altura em metros (m^2^). As circunferências da cintura (CC) e do quadril (CQ) foram obtidas por fita métrica de fibra de vidro flexível e inextensível. A CC, em centímetros (cm), foi medida em pé, no ponto médio entre a última costela e a crista ilíaca, na expiração. A CQ, em cm, aferida no ponto mais largo sobre o quadril/nádegas, com a fita paralela ao chão. Razão cintura/quadril (RCQ) e cintura/estatura (RCE) foram calculadas.

A composição corporal foi avaliada por Bioimpedância elétrica (BIA) para estimativa do percentual de gordura corporal (% gordura), método simples, prático e facilmente reproduzível. A BIA foi realizada através do *Biodynamics 450® analyzer* (*Biodynamics* Corp., Shoreline, WA, USA), frequência única de 50khz tetrapolar, em condições padronizadas.

### Análise bioquímica

Após jejum de 8 horas, foram coletadas amostras de sangue venoso e de urina. O hemograma foi medido por citometria de fluxo fluorescente, impedância e colorimetria, e realizado o cálculo da relação neutrófilos/linfócitos. Ácido úrico, glicose sérica, creatinina, colesterol total, colesterol de lipoproteína de alta densidade (HDL-c) foram medidos por análise automatizada *(Technicon DAX96, Miles Inc*). As concentrações de colesterol de lipoproteína de baixa densidade (LDL-c) foram calculadas pela equação de Friedewald. A insulina por radioimunoensaio e o *Homeostatic Model Assessment-Insulin Resistance* (HOMA-IR) foi utilizado para estimar a resistência à insulina. A determinação da concentração sérica de proteína C-reativa foi realizada pelo método turbidimétrico (látex de alta sensibilidade). Marcadores hepáticos foram analisados pelo método cinético ultravioleta. Eletrólitos como magnésio e cálcio pelo método colorimétrico. Tiroxina livre, hormônio estimulador da tireoide (TSH), paratormônio (PTH) e 25(OH)D foram analisados por imunoensaio eletroquimioluminescente. As amostras urinárias de cálcio foram analisadas por método colorimétrico de reação com cresolftaleína e da creatinina pelo método cinético, com posterior cálculo da relação cálcio-creatinina urinária.

### Avaliação da hemodinâmica central

Os parâmetros hemodinâmicos centrais foram acessados pelo Mobil-O Graph^®^ (I.E.M. GmbH, Stolberg, Germany). Um método não invasivo de análise da onda de pulso arterial por oscilometria, permitindo análise simultânea da pressão arterial (PA) periférica e central, da velocidade da onda de pulso (VOP) e do índice de incremento (Aix). A determinação da pressão arterial sistólica central (PASc) foi baseada na gravação das ondas de pulso da arterial, com insuflação do manguito por 10 segundos ao nível da PAD utilizando braçadeiras convencionais para adultos obesos e um sensor MPX50550 (*Freescale Inc., Tempe, AZ, EUA)*. PAS e pressão de pulso (PP) centrais e periféricas foram coletadas, e pressão de aumento (AP), Aix, Aix@75 (corrigido para frequência cardíaca 75bpm), VOP, amplificação de PP e idade arterial foram calculadas.

### Variabilidade da frequência cardíaca

A VFC foi utilizada para estimar a função autonômica através do frequencímetro (Polar^®^-*Verity Sense, Kempele, Finland*) posicionado e ajustado na face antero-medial do antebraço direito. Os indivíduos foram avaliados em posição sentada após cinco minutos de repouso, através da aferição denominada *ST (Short Term*), sendo validada para respiração normal em repouso.^17^ Todos os dados foram processados utilizando o *software* Kubios^®^ HRV (*Kubios Oy, Kuopio, Finland*) desprezando o primeiro minuto (período de relaxamento).

A cada batimento cardíaco (ondas R), os intervalos R-R (iRR) e outros parâmetros foram identificados e analisados quanto aos domínios do tempo e da frequência através de métodos lineares e não lineares. O domínio do tempo quantifica a VFC em milissegundos (ms) e reflete a atividade autonômica de maneira global. Utilizaram-se os parâmetros lineares: iRR - média dos intervalos entre cada batimento cardíaco; SDNN - desvio-padrão de todos os intervalos RR normais gravados em um intervalo de tempo, em ms; RMSSD - raiz quadrada da média do quadrado das diferenças entre os intervalos RR normais adjacentes, em um intervalo de tempo (ms). Na avaliação dos domínios de frequência (análise espectral), a menor variabilidade nos iRR tem mais relação com as bandas de menor frequência (LF- *low frequency*) e a maior variabilidade com as bandas de frequência mais elevada (HF*- high frequency*). Os métodos não lineares foram representados por um mapa de pontos em coordenadas cartesianas (*Poincaré plot*), indicando os índices SD1, desvio padrão da variabilidade instantânea batimento a batimento, e SD2, desvio padrão da variabilidade global da frequência cardíaca. O índice do sistema nervoso parassimpático (iSNP) foi calculado com a utilização da iRR, RMSSD e SD1, e do simpático (iSNS) através da FC média, índice de estresse (SI, de Baesvsky) e SD2. Ambos os algoritmos foram fornecidos pelo *software Kubios*^*®*^. As razões SD2/SD1 e LF/HF refletem o balanço simpatovagal.^18^

### Cálculo amostral

Para determinação do tamanho amostral deste estudo, a VOP foi considerada como desfecho primário. Desse modo para uma diferença mínima de 0,7m/s na VOP, desvio-padrão de 0,8 m/s, poder do estudo de 80% e nível de significância de 5%, seria necessário o mínimo de 44 pacientes. Considerando uma perda estimada de 10%, o número inicial previsto da amostra seria de 48 indivíduos.

### Análise estatística

As variáveis foram analisadas para normalidade pelo teste Shapiro–Wilk. As variáveis contínuas foram confirmadas com distribuição normal. O teste exato de Fisher foi utilizado para comparar variáveis categóricas. Os resultados foram apresentados como média ± desvio-padrão para as variáveis contínuas e em percentagem (%) para as variáveis categóricas. O Teste t pareado foi realizado para comparar as variáveis contínuas intragrupos entre S0 e S8. O Teste t de *Student* para amostras independentes foi utilizado para comparações entre variáveis contínuas intergrupos. A análise das correlações entre as variáveis foi realizada para obter o coeficiente de Pearson. A regressão linear múltipla foi utilizada para os ajustes de sexo e idade. Para todas as análises foi adotado o intervalo de confiança (IC) de 95% e o valor de p<0,05 considerado estatisticamente significativo. Os resultados foram obtidos com o auxílio do *software Statistical Package for Social Sciences*^*®*^ (SPSS, Chicago, Illinois, USA) versão 25.0.

## Resultados

A avaliação dos parâmetros clínicos, antropométricos e da composição corporal no período basal do estudo são apresentados na tabela 1 e os dados laboratoriais na tabela 2. A distribuição dos indivíduos nos dois grupos (CO, n=25 e VD, n=28) foi homogênea em S0, especialmente em relação à idade, IMC e níveis de vitamina D.

**Tabela 1:** – Variáveis clínicas e antropométricas dos grupos controle (CO) e grupo intervenção (VD), no período basal

Variáveis	CO (n=25)	VD (n=28)	p
Idade, anos	51±6	52±6	0,509
Sexo feminino (%)	51,2	48,8	0,337
Pacientes hipertensos (%)	41,2	58,8	0,572
Índice de Massa Corporal, kg/m^2^	30,9±3,7	31,3±3,7	0,760
Circunferência da cintura, cm	102±12	104±10	0,485
Razão cintura-quadril	0,89±0,09	0,90±0,08	0,596
Razão cintura-estatura	0,63±0,06	0,63±0,05	0,986
Gordura Corporal Total, %	38±4	37±7	0,309

Dados expressos em média±desvio padrão. P-valor: teste t independente para variáveis numéricas e teste exato de Fisher para variáveis categóricas.

**Tabela 2:** – Variáveis laboratoriais dos grupos controle (CO) e grupo intervenção (VD), no período basal

Variáveis	CO (n=25)	VD (n=28)	p
Relação Neutrófilo/Linfócito	1,94±0,73	2,07±1,14	0,617
Glicose, mg/dl	84±8	86±14	0,448
Insulina, µU/ml	12±7	13±6	0,381
HOMA-IR	2,39±1,39	2,87±1,60	0,252
Creatinina, mg/dl	0,94±0,16	0,92±0,22	0,720
Ácido úrico, mg/dl	4,3±1,2	4,8±1,5	0,116
Magnésio, mg/dl	2,1±0,18	2,0±0,2	0,157
Colesterol total, mg/dl	209±37	210±45	0,942
HDL-colesterol, mg/dl	60±16	51±16	0,043
LDL-colesterol, mg/dl	131±31	127±45	0,718
T4 livre, ng/ml	1,25±0,19	1,28±0,23	0,578
Hormônio estimulante da tireoide, µUI/ml	3,2±3,4	2,5±1,7	0,330
Aspartato aminotransferase, U/L	23±7	23±6	0,746
Alanina aminotransferase, U/L	23±12	27±12	0,140
Gama-glutamil transferase, U/L	34±23	56±85	0,220
Fosfatase alcalina, U/L	205±70	218±60	0,451
Proteína C-reativa, mg/dl	0,39±0,38	0,74±0,91	0,095
Vitamin D, ng/ml	22,3±5,6	21,5±4,6	0,590

Dados expressos em média±desvio padrão. P-valor: teste t independente. HOMA-IR: Homeostasis model assessment insulin resistence; HDL: lipoproteína de alta densidade; LDL: lipoproteína de baixa densidade; T4: fração livre hormônio tiroxina.

**Figura 2:**
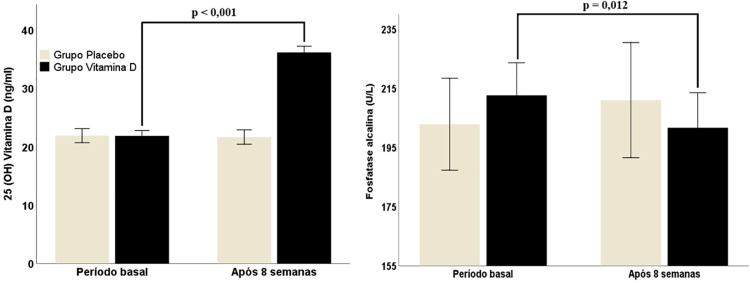
– Níveis séricos de vitamina D e fosfatase alcalina no período basal e após 8 semanas de intervenção.

**Figura 3:**
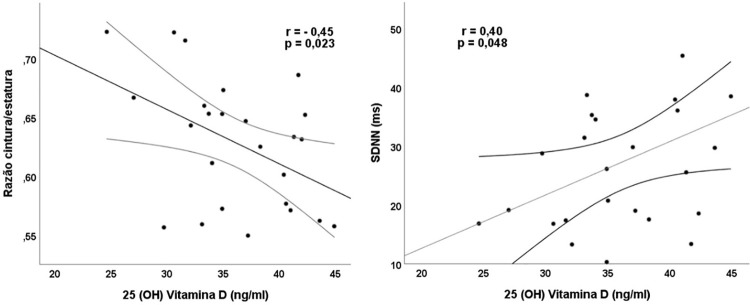
– Correlações da vitamina D com razão cintura/estatura e SDNN (desvio-padrão de todos os intervalos RR normais gravados em um intervalo de tempo) no grupo intervenção na oitava semana.

A Tabela 3 fornece os dados do PTH, cálcio sérico e corrigido pela albumina e a relação cálcio/creatinina urinaria nos momentos basal e pós-intervenção. Apenas no grupo VD, após 8 semanas de intervenção, houve aumento significativo nos níveis de vitamina D sérica e redução da fosfatase alcalina (Figura 2). Além disso, foram encontradas correlações inversas dos níveis de vitamina D com a RCE (Figura 3) e com o %gordura (r=-0,41 e p=0,044) mantendo significância estatística após os ajustes para sexo e idade (%gordura *β*=-0,914, IC-95% = 1,129/-0,099, p=0,029.

**Tabela 3:** – Níveis de paratormônio, cálcio sérico, cálcio corrigido pela albumina e relação cálcio/creatinina urinaria nos períodos basal e após 8 semanas de intervenção

Variáveis	Grupo Controle			Grupo Vitamina D		
	
	S0	S8	p	S0	S8	p
Paratormônio,pg/ml	60±23	56±23	0,455	65±23	66±32	0,758
Cálcio, mg/dl	9,8±0,3	9,8±0,3	0,705	9,9±0,6	9,8±0,4	0,186
Cálcio corrigido, mg/dl	9,6±0,3	9,6±0,3	0,694	9,8±0,5	9,6±0,4	0,251
Cálcio/creatinina urinária	0,09±0,08	0,09±0,09	0,784	0,09±0,08	0,07±0,05	0,194

Dados expressos em média±desvio padrão. P-valor: teste t pareado.

Na S8, houve redução significativa das PAS e PAM periféricas apenas no grupo VD. Com relação à hemodinâmica central, na S8, o grupo controle mostrou elevação significativa na AP e no Aix, não observado no grupo VD (tabela 4). Estes índices apresentaram correlações com outros parâmetros da hemodinâmica central como PPc (r=0,65, p=0,002 e r=0,51, p=0,015), VOP (r=0,65, p=0,002 e r=0,45, p=0,036) e com a idade vascular (r=0,62, p=0,003 e r=0,45, p=0,035), mas perderam significância estatística após ajustes para sexo e idade.

**Tabela 4:** Parâmetros hemodinâmicos periféricos e centrais nos períodos basal e após 8 semanas de intervenção

Variáveis	Grupo Controle			Grupo Vitamina D		
	
	S0	S8	p	S0	S8	p
**Hemodinâmica Periférica**						
PAS, mmHg	123±11	120±14	0,219	123±15	119±14	0,019
PAD, mmHg	84±7	83±8	0,353	83±9	81±8	0,068
PAM, mmHg	97±8	95±9	0,220	97±11	93±9	0,035
PP, mmHg	38±8	37±10	0,305	40±9	38±9	0,072
FC, bpm	70±10	73±12	0,112	68±10	67±11	0,520
**Hemodinâmica Central**						
PASc, mmHg	116±11	117±10	0,465	118±12	117±12	0,386
PPc, mmHg	30±8	30±5	0,852	31±7	32±6	0,583
Amplificação PP, %	30±10	32±7	0,505	27±12	24±12	0,311
AP, mmHg	9±5	12±8	0,028	8±5	8±5	0,748
Aix, %	26±12	35±19	0,020	26±14	25±14	0,827
Aix@75, %	23±10	24±9	0,502	21±13	15±12	0,053
VOP, m/s	7,2±0,9	7,3±0,8	0,386	7,5±0,93	7,4±0,8	0,255
Idade vascular, anos	50±8	51±7	0,329	53±8	52±7	0,558

Dados expressos em média±desvio padrão. P-valor: teste t pareado. PAS: pressão arterial sistólica; PAD: pressão arterial diastólica; PAM: pressão arterial média; PP: pressão de pulso; FC: frequência cardíaca; PASc: pressão arterial sistólica central; PPc: pressão de pulso central; AP: pressão de aumento; Aix: índice de incremento; Aix@75: índice de incremento corrigido para frequência cardíaca de 75 batimentos por minuto; VOP: velocidade da onda de pulso.

A avaliação da atividade autonômica na S8 mostra que o grupo VD apresentou um aumento no iSNP, no iRR, concomitante à redução na frequência cardíaca, no iSNS e no SI, sem alterações significativas no grupo controle (Tabela 5). O índice SDNN correlacionou-se positivamente com os níveis de vitamina D, mantendo significância estatística após ajustes para sexo e idade, obtidos por regressão linear múltipla (Figura 3).

**Tabela 5:** – Variabilidade da frequência cardíaca nos períodos basal e após 8 semanas de intervenção

Variáveis	Grupo Controle			Grupo Vitamina D		
	
	S0	S8	p	S0	S8	p
FC	72±13	73±11	0,934	71±11	67±11	0,046
iRR, ms	852±143	847±127	0,858	866±138	924±161	0,026
SDNN	39±36	29±12	0,115	24±12	27±12	0,253
RMSSD	39±46	29±16	0,206	25±13	31±16	0,102
Índice SNP	-0,37±1,35	-0,63±0,97	0,073	-0,64±0,94	-0,16±1,10	0,028
Índice SNS	1,08±1,37	1,23±1,26	0,519	1,68±2,02	0,92±1,53	0,033
SI	14±6	15±4	0,294	19±9	15±6	0,037
LF, ms^2^	614±999	332±263	0,213	320±388	336±315	0,860
HF, ms^2^	483±524	356±334	0,094	269±236	386±376	0,149
LF / HF	2,03±2,06	1,51±1,56	0,261	1,69±1,66	1,44±1,57	0,507
SD2/SD1	2,03±0,75	1,89±0,59	0,290	1,79±0,59	1,73±1,15	0,808

Dados expressos em média±desvio padrão. P-valor: teste t pareado. FC: frequência cardíaca; iRR: média dos intervalos entre cada batimento cardíaco; SDNN: desvio-padrão de todos os intervalos RR normais gravados em um intervalo de tempo (ms); RMSSD: raiz quadrada da média do quadrado das diferenças entre os intervalos RR normais adjacentes (ms); SNP: sistema nervoso parassimpático; SNS: sistema nervoso simpático; SI: índice de estresse; LF: componente de baixa frequência (low frequency); HF: componente de alta frequência (high frequency); SD2: desvio-padrão da variabilidade global da frequência cardíaca no gráfico de Poincaré e SD1: desvio-padrão da variabilidade instantânea batimento-a-batimento no gráfico Poincaré.

## Discussão

Os efeitos da suplementação de doses elevadas de vitamina D sobre diversos marcadores clínicos, bioquímicos e vasculares foram avaliados após dois meses em adultos com excesso de peso. Os dois grupos de estudo apresentaram semelhança quanto à idade, IMC elevado e níveis baixos de vitamina D, corroborando relatos de uma metanálise que descreve uma correlação inversa entre IMC e estado de vitamina D.^19^ No presente estudo, com a elevação nos níveis séricos da 25(OH)D, houve redução nos níveis pressóricos periféricos e aumento no tônus parassimpático.

Estudos prévios mostram uma relação inversa e significativa entre vitamina D e fosfatase alcalina, e a elevação desta pode sugerir remodelação óssea contínua naqueles indivíduos com baixos níveis séricos de vitamina D.^20^ O grupo intervenção apresentou redução dos níveis séricos de fosfatase alcalina, sugerindo recuperação de matriz óssea. A literatura relaciona os baixos níveis séricos de vitamina D com índices antropométricos elevados e, esses parâmetros quando avaliados após suplementação, apresentam resultados bastante heterogêneos, como relatado recentemente por Musazadeh et al. Esta metanálise observou que a vitamina D reduziu efetivamente a CC, porém sem afetar a gordura corporal. Os resultados do presente estudo são parcialmente semelhantes ao correlacionar inversamente os níveis de vitamina D com uma melhor RCE e %gordura corporal, sem, entretanto, afetá-los significativamente, muito provavelmente pelo curto período de suplementação.^21^

Apesar da forte associação de risco cardiovascular com deficiência grave (com morte súbita e acidente vascular cerebral) e moderada (com hipertensão e síndrome metabólica) da vitamina D, não há recomendação para sua utilização para prevenção de DCV, nem para tratamento da obesidade.^22^ Tal fato se deve aos resultados heterogêneos e muitas vezes inconsistentes dos estudos de suplementação e dos grandes ensaios, além do risco aumentado de toxicidade por suplementação com doses elevadas por períodos prolongados. Os riscos de hipercalcemia e hipercalcinose podem levar a diversos efeitos colaterais e precisam ser identificados. Neste estudo, a escolha de doses elevadas de suplementação da vitamina D foi realizada pela maior necessidade já demonstrada no excesso de peso. Entretanto, o período curto da intervenção funciona como proteção aos possíveis efeitos colaterais. Apenas a vitamina D sérica apresentou elevação significativa, sem atingir 100ng/ml (hipervitaminose) ou 150ng/ml (tóxicos).^22^ Não houve queixas clínicas dos indivíduos ou achados laboratoriais que pudessem ser relacionados com toxicidade como hipercalcemia, hipercalciúria ou redução importante dos níveis de PTH, garantindo a segurança dos sujeitos submetidos à intervenção.

Os estudos com suplementação de vitamina D encontram resultados variados quanto aos efeitos nas PA periféricas e centrais. Neste ensaio, a redução nas pressões periféricas se assemelha aos resultados de uma metanálise que incluiu ECRs, com suplementação superior a três meses e doses médias de vitamina D acima de 2000 UI/dia. Destes, seis estudos encontraram reduções na PA sistólica.^23^ Esta discreta redução remete ao estudo de Hardy et al., que relatam uma redução de 1 mmHg na pressão arterial sistólica sendo associada a menos eventos de insuficiência cardíaca.^24^

Ao término do presente estudo, o grupo controle evoluiu com aumento significativo nas AP e Aix, medidas indiretas de rigidez arterial, refletindo uma maior velocidade na propagação da onda refletida ao coração, achados não observados naqueles que receberam vitamina D, sugerindo um potencial efeito protetor nestes indivíduos, em relação ao envelhecimento vascular. Estes resultados não são semelhantes aos referidos em uma metanálise onde, entre 12 ECRs avaliados, nenhum apresentou piora do Aix nos grupos controle. Os efeitos protetores sobre a hemodinâmica central, após suplementação de vitamina D, ainda não podem ser afirmados pela heterogeneidade dos resultados, mas há relatos de redução no Aix em dois estudos, em populações de idosos e diabéticos tipo 2.^25^ Em parte do estudo *DAYLIGHT* (*Vitamin D Therapy in Individuals with Prehypertension or Hypertension)*, os autores encontraram reduções significativas na AP e no Aix em 41 indivíduos pré-hipertensos com IMC médio semelhante ao desta população, suplementando 4mil UI/dia de vitamina D por seis meses.^26^

A suplementação de vitamina D propiciou elevação do tônus parassimpático e concomitante redução no tônus simpático, demonstrados pelo aumento na VFC. De uma forma geral, a VFC reflete as alterações adaptativas do sistema nervoso autônomo, refletindo sua influência sobre o nó sinusal. O aumento da VFC sugere boa adaptação fisiológica pressupondo bom funcionamento cardiovascular. Uma VFC reduzida pode ser um indicador de má adaptação tanto por hiperatividade simpática ou em decorrência de uma retirada vagal, encontrada em alguns processos patológicos do sistema cardiovascular. O tônus parassimpático é demonstrado predominantemente pelo RMSSD, HF, e SD1, além do iPNS. Por outro lado, SDNN, LF, SD2, iSNS e SI são influenciados por efeito adrenérgico e colinérgico.^17^

A redução na VFC e a hipovitaminose D estão associadas a DCV e poucos estudos investigaram essa associação e os efeitos dessa suplementação. Recentemente uma revisão sobre micronutrientes chama a atenção para associação entre deficiência nos níveis de vitamina B12 e de Vitamina D com menor VFC.^27^ Tak et al. observaram uma correlação positiva entre os níveis de 25(OH)D e o SDNN, em indivíduos saudáveis, usando registros de intervalo RR de 5 minutos, semelhantes aos desse estudo.^28^ Há relatos de outros dois estudos transversais, também em indivíduos saudáveis, mostrando associação entre baixos níveis de vitamina D e redução na VFC, utilizando avaliação distinta (gravações de 24 horas).^29,30^ Também foi demonstrada redução na VFC em dois ECRs em pacientes diabéticos com disautonomia cardíaca e em um estudo em indivíduos com insuficiência cardíaca crônica.^31-33^

Em 2016, Mann et al. realizaram o primeiro estudo de suplementação de vitamina D objetivando melhora na adaptação autonômica, em pacientes saudáveis e com baixos níveis de vitamina D. Uma resposta protetiva cardiovagal por elevação no componente HF foi encontrada nos indivíduos suplementados, após estímulo estressor (injeção intravenosa de angiotensina II por 30 minutos). Pré suplementação, a resposta autonômica era desfavorável com elevação significativa da relação LF:HF.^34^ Dogdus et al. encontraram aumento na VFC em adultos saudáveis com baixos níveis de vitamina D após suplementação em dose única, avaliada por eletrocardiograma de 24h.^35^

O ensaio *VITAH (Vitamin D supplementation and cardiac autonomic tone in hemodialisys)* postulou se a suplementação intensiva (50.000 UI/semana) de vitamina D reduziria a relação LF:HF, quando adicionada à suplementação básica já em uso para pacientes em hemodiálise (alfacalcidol 0,25mcg 3 vezes/semana). Após a intervenção intensiva, não foi verificado redução na relação LF:HF. Entretanto, só houve elevação nesta relação nos indivíduos que permaneceram com vitamina D sérica insuficiente, mesmo após suplementação intensiva por seis semanas.^36^ De forma semelhante, este ensaio encontrou proteção vagal após a suplementação da vitamina D, pela elevação significativa do tônus parassimpático. Adicionalmente, esses achados se corroboraram, de forma consistente, pela redução significativa do tônus simpático.

O presente estudo apresenta algumas limitações. A amostra populacional pode ser considerada pequena, mas atingiu o tamanho amostral mínimo calculado previamente, o que foi suficiente para obter significância estatística na análise comparativa de algumas variáveis entre os grupos. Além disso, os métodos de estudo da estrutura vascular foram mais específicos do que aqueles realizados em outros estudos com vitamina D, o que permitiu um tamanho amostral mais reduzido. O tempo de estudo também pode ser considerado insuficiente para obter resultados mais relevantes. Por outro lado, os achados significativos observados com apenas oito semanas de intervenção reforçam os efeitos precoces da suplementação de vitamina D e trazem mais originalidade para o presente estudo.

## Conclusão

Nesta amostra de indivíduos com sobrepeso ou obesidade, a suplementação diária de vitamina D durante oito semanas resultou em melhora das pressões periféricas e do equilíbrio autonômico. Houve manutenção das pressões centrais sugerindo atenuação da evolução clínica desfavorável observada na ausência desta reposição. Estes resultados positivos em relação aos parâmetros hemodinâmicos e proteção autonômica se tornam relevantes justamente pelo curto período de intervenção e pela escolha da população com excesso de peso, concomitante aos baixos níveis de vitamina D. Esses achados reforçam a hipótese de um efeito protetor da vitamina D no sistema cardiovascular em algumas situações clínicas e indicam a necessidade de novos estudos randomizados em maior escala e por período prolongado visando maior nível de evidência.
