# Supplementary material for: Efeitos da Suplementação de Vitamina D sobre Parâmetros Hemodinâmicos Centrais e Sistema Nervoso Autônomo em Indivíduos com Obesidade ou Sobrepeso
Source: Arq Bras Cardiol. 2024 Apr 26;121(5):e20230678. [Article in Portuguese] doi: 10.36660/abc.20230678 (PMC11098585; doi:10.36660/abc.20230678)
Supplement: Translation

# Translation: Effects of Vitamin D Supplementation on Central Hemodynamic Parameters and Autonomic Nervous System in Obese or Overweight Individuals

**DOI:** 10.36660/abc.20230678i

**Authors:** Adriana C. C. Faria, Caroline Lyra Moreira, Michelle Rabello da Cunha, Samanta Mattos, Wille Oigman, Mario Fritsch Neves

**Affiliations:** 1 Brazil Departamento de Clínica Médica, Universidade do Estado do Rio de Janeiro, Rio de Janeiro, RJ – Brazil; 2 Brazil Departamento de Nutrição Aplicada, Instituto de Nutrição, Universidade do Estado do Rio de Janeiro, Rio de Janeiro, RJ – Brazil

**Keywords:** Vitamin D, Autonomic Nervous System, Hemodynamics

## Abstract

**Background:**

Previous studies have been inconsistent in demonstrating beneficial cardiovascular effects of vitamin D supplementation.

**Objective:**

To evaluate the effects of vitamin D3 supplementation on central hemodynamic parameters and autonomic activity in obese/overweight individuals with low vitamin D levels (<30ng/dl).

**Methods:**

Adults 40-65 years old with body mass index ≥25<40 kg/m^2^ were enrolled in this prospective, randomized, double-blind clinical trial (NCT 05689632). Central hemodynamics was assessed using the oscillometric method (Mobil-O-Graph^®^), and heart rate variability using a Polar heart rate monitor (Kubios^®^ software). Patients (n=53) received a placebo in the control group (CO, n=25) or vitamin D3 (VD, n=28) 7000 IU/day, and were evaluated before (W0) and after 8 weeks (W8) with a significance level of 0.05.

**Results:**

The groups were homogeneous regarding age (51±6 vs 52±6 years, p=0.509) and vitamin D levels (22.8±4.9 vs 21.7±4.5ng/ml, p=0.590). At W8, the VD group had significantly higher levels of vitamin D (22.5 vs 35.6ng/ml, p<0.001). Only the VD group showed a significant reduction in systolic blood pressure (SBP; 123±15 vs 119±14mmHg, p=0.019) and alkaline phosphatase (213±55 vs 202±55mg/dl, p=0.012). The CO group showed an increase in augmentation pressure (AP: 9 vs 12 mmHg, p=0.028) and augmentation index (AIx: 26 vs 35%, p=0.020), which was not observed in the VD group (AP: 8 vs 8 mmHg, AIx: 26 vs 25%, p>0.05). VD group showed an increase in the parasympathetic nervous system index (PNSi) (-0.64±0.94 vs -0.16±1.10, p=0.028) and the R-R interval (866±138 vs 924±161 ms, p= 0.026).

**Conclusion:**

In this sample, eight weeks of daily vitamin D supplementation resulted in an improvement in blood pressure levels and autonomic balance.

## Introduction

Obesity directly contributes to a higher cardiovascular risk with increased mortality, independently of other risk factors. Several mechanisms can modulate this adverse effect since there is considerable metabolic and inflammatory heterogeneity between obese and overweight individuals.^1-3^ Vitamin D is a steroid hormone essential for the regulation of skeletal health, and concentrations of 25-hydroxyvitamin D (25(OH)D) between 20ng/ml and 50ng/ml (50–125 nmol/l) have been considered safe for the maintenance of skeletal health in the general population.^4,5^ In recent years, additional functional roles of vitamin D (pleiotropic effects) have been evidenced, linking its deficiency (25(OH)D < 20ng/dl) and suboptimal state (25(OH)D ≥ 20 and <30ng/dl) to several diseases and unfavorable metabolic factors, such as insulin resistance, type 2 diabetes mellitus and cardiovascular diseases (CVD).^6,7^ Epidemiological studies have shown that vitamin D deficiency is associated with an increased risk of future cardiovascular events and arterial stiffness.^8,9^

The difficulty in maintaining adequate vitamin D levels is highly prevalent among overweight individuals, probably due to volumetric dilution (lipossolubility) and adipose tissue sequestration (reservoir) among other hypotheses.^10,11^ The response to vitamin D supplementation is lower in these individuals, indicating dose adjustments to body size.^12^ Obesity and vitamin D deficiency together promote inflammation and induce vascular dysfunction through the increased release of vasoconstriction factors, which are harmful to vascular inflammation and endothelial activation. Both are associated with worse cardiovascular health and are considered risk factors for vascular and metabolic changes and worse clinical outcomes.^13,14^

Observational studies support the physiological theory of vitamin D supplementation improving vascular parameters and clinical events.^15^ However, results from selected randomized clinical trials (RCTs) have been inconsistent regarding this protective effect. Some factors, such as heterogeneous populations and low supplementation doses incapable of altering the previous vitamin D status, may be related to these failures.^14^ Therefore, the decision to enroll only deficient or insufficient vitamin D serum levels subjects and the use of high and safe doses of Vitamin D supplementation adopted in this study follows recently described criteria stipulated for carrying out studies with nutrients.^16^

To date, no studies have been observed aiming to improve heart rate variability (HRV) in patients with low levels of vitamin D and overweight. The objective of this study was to evaluate the effects of vitamin D supplementation on the cardiovascular system by assessing arterial stiffness and autonomic nervous system activity.

## Methods

### Subjects

Adult volunteers, of both genders, aged between 40 and 70 years, body mass index (BMI) between 25.0 and 39.9 kg/m^2^ and 25(OH)D levels <30 ng/ml were enrolled. Exclusion criteria were diabetes mellitus, peripheral arterial, coronary, or chronic kidney diseases, use of beta-blockers or supplements containing vitamin D, pregnancy, or cancer in the last 5 years. The protocol was approved by the local Research Ethics Committee (CAAE: 61044522.0.0000.5259) and all participants read and signed the participatory and informed consent (PIC). This study was carried out following resolution nº 466/2012 and was registered at clinicaltrials.gov (NCT 05689632).

### Study design and randomization

A prospective, randomized, double-blind, placebo-controlled clinical trial was carried out at the Clinic of Hypertension and Associated Metabolic Diseases, Pedro Ernesto University Hospital, Rio de Janeiro State University, Brazil. Figure 1 presents the study flowchart with 53 participants randomly assigned, according to the randomization plan (wwww.randomization.com) to receive a placebo (medium chain triglycerides (MCT) 100mg) in the control group (CO, n=25) or Vitamin D3 7.000UI and TCM 100mg in the intervention group (VD, n=28), daily after lunch, for 8 weeks. The trial was double-blind, for the team and volunteers, who received unlabeled, numbered bottles, containing the same number of capsules identical in color, shape, and size. All capsules were manufactured in the same unit (Centralfarma – Ipatinga, Minas Gerais, Brazil). Adherence was assessed by the percentage of capsules consumed and an adherence rate of 80% was satisfactorily guaranteed. All exams were performed before (W0) and after 8 weeks (W8) of intervention.

**Figure 1:** – Study flowchart.

### Blood pressure, anthropometric, and body composition assessment

Measurements of systolic (SBP) and diastolic (DBP) blood pressure, calculation of mean arterial pressure (MAP), and heart rate (HR) were obtained using a calibrated digital device (model HEM-705 CP, OMRON Healthcare Inc., Illinois), in a sitting position, on the right upper limb, after 5 minutes of rest.

Weight was measured on a digital scale with an accuracy of ± 0.1 kg (Filizola SA, São Paulo, SP, Brazil) and BMI was calculated using the formula: weight in kilograms divided by the square of height in meters (m^2^). Waist (WC) and hip (HC) circumferences were obtained using a flexible and inextensible fiberglass measuring tape. WC was measured standing up, at the midpoint between the last rib and the iliac crest, during expiration. The HC was measured at the widest point on the hip/buttocks, with the tape parallel to the floor. Waist/hip ratio (WHR) and waist/height ratio (WHtR) were calculated.

Body composition was assessed using Bioelectrical Impedance (BIA) to estimate the percentage of body fat (% fat), a simple, practical, and easily reproducible method. BIA was performed using the Biodynamics 450® analyzer (Biodynamics Corp., Shoreline, WA, USA), single frequency of 50 kHz tetrapolar, under standardized conditions.

### Biochemical analysis

After 8 hours of fasting, venous blood and urine samples were collected. The blood count was measured by fluorescent flow cytometry, impedance, and colorimetry, and the neutrophil/lymphocyte ratio was calculated. Uric acid, serum glucose, creatinine, total cholesterol, and high-density lipoprotein cholesterol (HDL-c) were measured by automated analysis (Technicon DAX96, Miles Inc). Low-density lipoprotein cholesterol (LDL-c) concentrations were calculated using the Friedewald equation. Radioimmunoassay insulin and Homeostatic Model Assessment-Insulin Resistance (HOMA-IR) were used to estimate insulin resistance. The determination of the serum concentration of C-reactive protein was carried out using the turbidimetric method (high-sensitivity latex). Liver markers were analyzed using the ultraviolet kinetic method. Electrolytes such as magnesium and calcium using the colorimetric method. Free thyroxine, thyroid-stimulating hormone (TSH), parathyroid hormone (PTH), and 25(OH)D were analyzed by electrochemiluminescent immunoassay. Urinary calcium samples were analyzed using the colorimetric method of reaction with cresolphthalein and creatinine using the kinetic method, with subsequent calculation of the urinary calcium-creatinine ratio.

### Central hemodynamics assessment

Central hemodynamic parameters were accessed by Mobil-O Graph^®^ (I.E.M. GmbH, Stolberg, Germany). A non-invasive, oscillometric method of arterial pulse wave analysis, allows simultaneous assessment of peripheral and central blood pressure (BP), pulse wave velocity (PWV), and augmentation index (AIx). The determination of central systolic blood pressure (cSBP) was based on the recording of arterial pulse waves, with cuff inflation for 10 seconds at the level of DBP using conventional cuffs for obese adults and an MPX50550 sensor (Freescale Inc., Tempe, AZ, USA). Central and peripheral SBP and pulse pressure (PP) were collected, and augmentation pressure (AP), AIx, AIx@75 (adjusted for heart rate 75bpm), PWV, PP amplification, and arterial age were calculated.

### Heart rate variability

HRV was used to estimate autonomic function using a heart rate monitor (Polar^®^-Verity Sense, Kempele, Finland) positioned and adjusted on the anteromedial surface of the right forearm. The individuals were evaluated in a sitting position after five minutes of rest, using the measurement called ST (Short Term), which is validated for normal breathing at rest.^17^ All data were processed using the Kubios^®^ HRV software (Biosignal Analysis and Medical Imaging Group, Kuopio, Finland) disregarding the first minute (relaxation period).

At each heartbeat (R waves), the mean R-R intervals (mRR) and other parameters were identified and analyzed in the time and frequency domains using linear and non-linear methods. The time domain quantifies HRV in milliseconds (ms) and reflects autonomic activity globally. The following linear parameters were calculated: mRR - average of the intervals between each heartbeat; SDNN - standard deviation of all normal RR intervals recorded in a time interval, in ms and RMSSD - square root of the mean of the square of the differences between adjacent normal RR intervals, in a time interval (ms). When evaluating frequency domains (spectral analysis), the lower variability in iRR is more related to the lower frequency bands (LF- low frequency) and the greater variability to the higher frequency bands (HF- high frequency). The non-linear methods were represented by a map of points in Cartesian coordinates (Poincaré plot), indicating the indices SD1, the standard deviation of instantaneous beat-to-beat variability, and SD2, the standard deviation of global heart rate variability. The parasympathetic nervous system index (PNSi) was calculated using mRR, RMSSD, and SD1, and the sympathetic nervous system index (SNSi) using mean HR, stress index (SI, Baesvsky), and SD2. Both algorithms were provided by Kubios^®^ software. The SD2/SD1 and LF/HF ratios reflect the sympathovagal balance.^18^

### Sample size calculation

To determine the sample size for this study, PWV was considered as the primary outcome. Therefore, for a minimum difference of 0.7 m/s in PWV, a standard deviation of 0.8 m/s, a study power of 80%, and a significance level of 5%, a minimum of 44 patients would be necessary. Considering an estimated loss of 10%, the expected initial number of the sample would be 48 individuals.

### Statistical analysis

Variables were analyzed for normality using the Shapiro–Wilk test. Continuous variables were confirmed with normal distribution. Fisher’s exact test was used to compare categorical variables. The results are presented as mean ± standard deviation for continuous variables and as a percentage (%) for categorical variables. Paired t-test was performed to compare intragroup continuous variables between W0 and W8. Student’s t-test for independent samples was used for comparisons between continuous intergroup variables. The analysis of correlations between variables was carried out to obtain the Pearson coefficient. Multiple linear regression was used to adjust for sex and age. For all analyses, the 95% confidence interval (CI) was adopted and the p-value <0.05 was considered statistically significant. The results were obtained with the aid of the Statistical Package for Social Sciences® (SPSS, Chicago, Illinois, USA) software version 25.0.

## Results

The evaluation of clinical, anthropometric, and body composition parameters in the baseline period of the study are presented in Table 1, and laboratory data in Table 2. The individuals were evenly distributed between the two groups (CO, n=25 and VD, n=28) in W0, especially according to age, BMI, and vitamin D levels.

**Table 1:** – Baseline clinical and anthropometric variables of control group (CO) and intervention group (VD)

Variables	CO (n=25)	VD (n=28)	p
Age, years	51±6	52±6	0.509
Female, n (%)	51.2	48.8	0.337
Previous hypertension, n (%)	41.2	58.8	0.572
Body Mass Index, kg/m^2^	30.9±3.7	31.3±3.7	0.760
Waist circunference, cm	102±12	104±10	0.485
Waist-to-hip ratio	0.89±0.09	0.90±0.08	0.596
Waist-to-height ratio	0.63±0.06	0.63±0.05	0.986
Body fat, (%)	38±4	37±7	0.309

Data are expressed as mean ± SD or proportions. P value corresponds to the Student t-test for continuous variables and Fisher’s exact test for categorical variables.

**Table 2:** – Baseline biochemical analysis of the control group (CO) and intervention group (VD)

Variables	CO (n=25)	VD (n=28)	p
Neutrophil to lymphocyte ratio	1.94±0.73	2.07±1.14	0.617
Glucose, mg/dl	84±8	86±14	0.448
Insulin, µU/ml	12±7	13±6	0.381
HOMA-IR	2.39±1.39	2.87±1.60	0.252
Creatinine, mg/dl	0.94±0.16	0.92±0.22	0.720
Uric acid, mg/dl	4.3±1.2	4.8±1.5	0.116
Magnesium, mg/dl	2.1±0.18	2.0±0.2	0.157
Total cholesterol, mg/dl	209±37	210±45	0.942
HDL-cholesterol, mg/dl	60±16	51±16	0.043
LDL-cholesterol, mg/dl	131±31	127±45	0.718
Free T4, ng/ml	1.25±0.19	1.28±0.23	0.578
TSH, µUI/ml	3.2±3.4	2.5±1.7	0.330
Aspartate transaminase, U/L	23±7	23±6	0.746
Alanine transaminase, U/L	23±12	27±12	0.140
GGT, U/L	34±23	56±85	0.220
Alkaline phosphatase, U/L	205±70	218±60	0.451
C-reactive protein, mg/dl	0.39±0.38	0.74±0.91	0.095
Vitamin D, ng/ml	22.3±5.6	21.5±4.6	0.590

Data are expressed as mean ± SD. p-value Student’s t-test. HOMA-IR: Homeostasis model assessment insulin resistance; HDL: high density lipoprotein; LDL: low density lipoprotein; T4: Free thyroxine; TSH: Thyroid-stimulating hormone; GGT: Gamma-glutamyl transferase.

Table 3 provides data on PTH, serum, and albumin-corrected calcium and the urinary calcium/creatinine ratio at baseline and post-intervention. Only in the VD group, after 8 weeks of intervention, there was a significant increase in serum vitamin D levels and a reduction in alkaline phosphatase (ALP) (Figure 2). Furthermore, inverse correlations were found between vitamin D levels and WHtR (Figure 3) and with %fat (r=-0.41 and p=0.044), maintaining statistical significance after adjustments for sex and age (%fat β= -0.914, 95% CI =-1.129/-0.099, p=0.029).

**Table 3:** – Baseline and post-intervention parathyroid hormone, calcium, albumin-corrected calcium, and the urinary calcium/creatinine ratio

Variables	Control Group			Vitamin D Group		
	
	W0	W8	p	W0	W8	p
PTH, pg/ml	60±23	56±23	0.455	65±23	66±32	0.758
Calcium, mg/dl	9.8±0.3	9.8±0.3	0.705	9.9±0.6	9.8±0.4	0.186
Albumin-corrected calcium, mg/dl	9.6±0.3	9.6±0.3	0.694	9.8±0.5	9.6±0.4	0.251
Urinary calcium/creatinine ratio	0.09±0.08	0.09±0.09	0.784	0.09±0.08	0.07±0.05	0.194

Data expressed as mean ± SD. P-value Student’s t-test. PTH: Parathyroid hormone.

**Figure 2:** – Baseline and post-intervention Vitamin D and alkaline phosphatase levels.

**Figure 3:** – Baseline and post-intervention correlations of Vitamin D with waist-to-height ratio and SDNN (standard deviation of all normal RR intervals).

At W8, there was a significant reduction in peripheral SBP and MAP only in the VD group. Regarding central hemodynamics at W8, the control group showed a significant increase in AP and AIx, not observed in the VD group (Table 4). These indices showed correlations with other central hemodynamic parameters such as cPP (r=0.65, p=0.002 and r=0.51, p=0.015), PWV (r=0.65, p=0.002 and r=0.45, p=0.036) and with vascular age (r=0.62, p=0.003 and r=0.45, p=0.035), but lost statistical significance after adjustments for sex and age.

**Table 4:** – Baseline and post-intervention peripheral and central hemodynamic parameters

Variables	Control Group			Vitamin D Group		
	
	W0	W8	p	W0	W8	p
**Peripheral Hemodynamics**						
SBP, mmHg	123±11	120±14	0.219	123±15	119±14	0.019
DBD, mmHg	84±7	83±8	0.353	83±9	81±8	0.068
MAP, mmHg	97±8	95±9	0.220	97±11	93±9	0.035
PP, mmHg	38±8	37±10	0.305	40±9	38±9	0.072
HR, bpm	70±10	73±12	0.112	68±10	67±11	0.520
**Central Hemodynamics**						
cSBP, mmHg	116±11	117±10	0.465	118±12	117±12	0.386
cPP, mmHg	30±8	30±5	0.852	31±7	32±6	0.583
PP amplification, %	30±10	32±7	0.505	27±12	24±12	0.311
AP, mmHg	9±5	12±8	0.028	8±5	8±5	0.748
Aix, %	26±12	35±19	0.020	26±14	25±14	0.827
Aix@75, %	23±10	24±9	0.502	21±13	15±12	0.053
PWV, m/s	7.2±0.9	7.3±0.8	0.386	7.5±0.93	7.4±0.8	0.255
Vascular age, years	50±8	51±7	0.329	53±8	52±7	0.558

Data expressed as mean ± SD. p-value Student’s t-test. SBP: systolic blood pressure; DBP: diastolic blood pressure; MAP: mean arterial pressure; PP: pulse pressure; HR: heart rate; cSBP: central systolic blood pressure; cPP: central pulse pressure; AP: augmentation pressure; Aix: Augmentation index; Aix@75: augmentation index adjusted for heart rate 75bpm; PWV: pulse wave velocity.

The assessment of autonomic activity at W8 shows that the VD group presented an increase in PNSi, and mRR, along with a reduction in heart rate, SNSi, and SI, without significant changes in the control group (Table 5). The SDNN index correlated positively with vitamin D levels, maintaining statistical significance after adjustments for sex and age, obtained by multiple linear regression (Figure 3).

**Table 5:** – Baseline and post-intervention heart rate variabilit

Variables	Control Group			Vitamin D Group		
	
	W0	W8	p	W0	W8	p
HR	72±13	73±11	0.934	71±11	67±11	0.046
mRR, ms	852±143	847±127	0.858	866±138	924±161	0.026
SDNN	39±36	29±12	0.115	24±12	27±12	0.253
RMSSD	39±46	29±16	0.206	25±13	31±16	0.102
PNSi	-0.37±1.35	-0.63±0.97	0.073	-0.64±0.94	-0.16±1.10	0.028
SNSi	1.08±1.37	1.23±1.26	0.519	1.68±2.02	0.92±1.53	0.033
SI	14±6	15±4	0.294	19±9	15±6	0.037
LF, ms^2^	614±999	332±263	0.213	320±388	336±315	0.860
HF, ms^2^	483±524	356±334	0.094	269±236	386±376	0.149
LF/HF	2.03±2.06	1.51±1.56	0.261	1.69±1.66	1.44±1.57	0.507
SD2/SD1	2.03±0.75	1.89±0.59	0.290	1.79±0.59	1.73±1.15	0.808

Data expressed as mean ± SD. p-value Student’s t-test. FC: heart rate; mRR: beat-to-beat RR interval mean; SDNN: standard deviation of all normal RR intervals; RMSSD: root mean square of successive differences between RR intervals; PNS: parasympathetic nervous system index; SNS: sympathetic nervous system index; SI: stress index; LF: low frequency; HF: high frequency; SD2: standard deviation of overall variability of the Poincare plot; SD1: standard deviation of instantaneous beat-to-beat variability of the Poincare plot.

## Discussion

The effects of high-dose vitamin D supplementation on several clinical, biochemical, and vascular markers were evaluated after two months in overweight adults. The two study groups were similar in terms of age, high BMI, and low levels of vitamin D, corroborating reports from a meta-analysis that describes an inverse correlation between BMI and vitamin D status.^19^ In the present study, with the increase in serum levels of 25(OH)D, there was a reduction in peripheral blood pressure levels and an increase in parasympathetic tone.

Previous studies show an inverse and significant relationship between vitamin D and alkaline phosphatase. Increases in ALP may suggest ongoing bone remodeling in those individuals with low serum levels of vitamin D.^20^ The intervention group showed a reduction in ALP levels, suggesting recovery of bone matrix. The literature links low serum levels of vitamin D with high anthropometric indices and, when these parameters are evaluated after supplementation, they present very heterogeneous results, as recently reported by Musazadeh et al. This meta-analysis observed that vitamin D effectively reduced WC without affecting body fat. The results of the present study are partially similar when shows vitamin D levels inversely correlated with better WHR and % body fat, without, however, significantly affecting them, most likely due to the short period of supplementation.^21^

Despite the strong association of cardiovascular risk with severe (with sudden death and stroke) and moderate (with hypertension and metabolic syndrome) vitamin D deficiency, there is no recommendation for its use to prevent CVD, nor for obesity treatment.^22^ This fact is due to the heterogeneous and often inconsistent results of supplementation studies and large trials, in addition to the increased risk of toxicity due to supplementation with high doses for prolonged periods. The risks of hypercalcemia and hypercalcinosis can lead to several side effects and need to be identified. In this study, the decision to administer high doses of vitamin D supplementation was based on previous evidence suggesting that overweight individuals may have a greater need for vitamin D. On the other hand, the short period of intervention works as a protection against possible side effects. Despite the high doses administered, serum vitamin D levels increased significantly but did not reach levels associated with hypervitaminosis (>100ng/ml) or toxicity (>150ng/ml).^22^ There were no clinical complaints from the individuals or laboratory findings that could be related to toxicity such as hypercalcemia, hypercalciuria, or significant reduction in PTH levels, ensuring the safety of subjects undergoing the intervention.

Vitamin D supplementation studies find several results regarding the effects on peripheral and central BP. This statement suggests that the reduction in peripheral pressures observed in the trial is consistent with the findings of a meta-analysis that included randomized controlled trials (RCTs) examining the effects of vitamin D supplementation over periods longer than three months and with average doses exceeding 2000 IU/day. Of these, six studies found reductions in systolic BP.^23^ This slight reduction refers to the study by Hardy et al. who reported a reduction of 1 mmHg in systolic blood pressure being associated with fewer heart failure events.^24^

At the conclusion of the study, the control group experienced a notable increase in AP and AIx, both of which are indirect measures of arterial stiffness. An elevated Aix indicates an increased speed at which the reflected wave from arterial branching returns to the heart during the cardiac cycle. However, in contrast, the group that received vitamin D did not exhibit these increases, suggesting a potential protective effect against vascular aging. This implies that vitamin D supplementation may have mitigated or slowed down the progression of arterial stiffness in these individuals.

On the other hand, the results of the present study contrast with those of a meta-analysis where none of the 12 RCTs evaluated showed worsening of AIx in the control groups. The protective effects on central hemodynamics after vitamin D supplementation cannot be confirmed due to the heterogeneity of the results, but there are reports of a reduction in AIx in specific populations such as elderly individuals and those with type 2 diabetes.^25^ In part of the DAYLIGHT study (Vitamin D Therapy in Individuals with Prehypertension or Hypertension), the authors found significant reductions in AP and AIx in 41 prehypertensive individuals with an average BMI similar to this population, supplementing 4.000 IU/day of vitamin D for six months.^26^

Vitamin D supplementation led to an increase in parasympathetic tone and a concomitant reduction in sympathetic tone, demonstrated by an increase in HRV. In general, HRV reflects the adaptive changes of the autonomic nervous system, reflecting its influence on the sinus node. The increase in HRV suggests good physiological adaptation, assuming good cardiovascular functioning. A reduced HRV may be an indicator of poor adaptation either due to sympathetic hyperactivity or as a result of vagal withdrawal, found in some pathological processes of the cardiovascular system. The parasympathetic tone is demonstrated predominantly by RMSSD, HF, and SD1, in addition to SNPi. On the other hand, SDNN, LF, SD2, SNSi, and SI are influenced by adrenergic and cholinergic effects.^17^

Reduced HRV and hypovitaminosis D are associated with CVD and few studies have investigated this association and the effects of this supplementation. Recently, a review on micronutrients drew attention to the association between deficiency in vitamin B12 and Vitamin D levels with lower HRV.^27^ Tak et al. observed a positive correlation between 25(OH)D levels and SDNN in healthy individuals, using 5-minute RR interval recordings, like those adopted in this study.^28^ There are reports of two other cross-sectional studies, also in healthy individuals, showing an association between low levels of vitamin D and a reduction in HRV, using different assessments (24-hour recordings).^29,30^ Reduction in HRV was also demonstrated in two RCTs in diabetic patients with cardiac dysautonomia and one study in individuals with chronic heart failure.^31-33^

In 2016, Mann et al. carried out the first vitamin D supplementation study aiming to improve autonomic adaptation in healthy patients with low vitamin D levels. A cardiovagal protective response due to an increase in the HF component was found in supplemented individuals, after a stressful stimulus (intravenous injection of angiotensin II for 30 minutes). Pre-supplementation, the autonomic response was unfavorable with a significant increase in the LF: HF ratio.^34^ Dogdus et al. found an increase in HRV in healthy adults with low levels of vitamin D after single-dose supplementation, assessed by 24-hour electrocardiogram.^35^

The VITAH (Vitamin D supplementation and cardiac autonomic tone in hemodialysis) trial postulated whether intensive vitamin D supplementation (50.000 IU/week) would reduce the LF: HF ratio when added to the basic supplementation already in use for hemodialysis patients (alfacalcidol 0.25mcg 3 times/week). After the intensive intervention, there was no reduction in the LF: HF ratio. However, this relationship only increased in individuals who remained with insufficient serum vitamin D, even after intensive supplementation for six weeks.^36^ Similarly, this trial found vagal protection after vitamin D supplementation, due to a significant increase in parasympathetic tone. Additionally, these findings were consistently corroborated by the significant reduction in sympathetic tone.

The present study has some limitations. The population sample can be considered small, but it reached the previously calculated minimum sample size, which was sufficient to obtain statistical significance in the comparative analysis of some variables between the groups. Furthermore, the methods for studying the vascular structure were more specific than those carried out in other studies with vitamin D, which allowed a smaller sample size. Study time can also be considered insufficient to obtain more relevant results. On the other hand, the significant findings observed after only eight weeks of intervention reinforce the early effects of vitamin D supplementation and bring more originality to the present study.

## Conclusion

In this sample of overweight or obese individuals, daily vitamin D supplementation for eight weeks resulted in improvements in peripheral pressures and autonomic balance. Central pressures were maintained, suggesting an attenuation of the unfavorable clinical evolution observed in the absence of this replacement. These positive results concerning hemodynamic parameters and autonomic protection become relevant precisely due to the short period of intervention and the choice of the overweight population, concomitant with low levels of vitamin D. These findings reinforce the hypothesis of a protective effect of vitamin D on the cardiovascular system in some clinical situations and indicate the need for new randomized studies on a larger scale and for a prolonged period aiming at a higher level of evidence.
